# Patient Portal Use and Risk of Readmissions in Decompensated Cirrhosis: Retrospective Study

**DOI:** 10.2196/47080

**Published:** 2023-12-19

**Authors:** Jeremy Louissaint, Jeffrey Gibbs, Abhishek Shenoy, Shirley Cohen-Mekelburg, Anna Lok, Elliot Tapper

**Affiliations:** 1 Division of Digestive and Liver Diseases, University of Texas Southwestern Dallas, TX United States; 2 Department of Internal Medicine, University of Michigan Ann Arbor, MI United States; 3 Institute for Healthcare Policy and Innovation, University of Michigan Ann Arbor, MI United States; 4 VA Center for Clinical Management Research, VA Ann Arbor Health Care System Ann Arbor, MI United States; 5 Gastroenterology Section, VA Ann Arbor Healthcare System Ann Arbor, MI United States

**Keywords:** cirrhosis, patient portal, readmissions, telehealth, telemedicine

## Abstract

**Background:**

Patient portals are a common electronic medical record tool that allow for the asynchronous exchange of health information between patients and their health care teams. Patients can leverage patient portals to perform tasks such as viewing test results, reviewing clinical notes, and messaging their health care team. The impact of patient portal use on clinical outcomes in cirrhosis is unknown.

**Objective:**

In this study, we evaluated the relationship between patient portal use patterns and readmissions in cirrhosis.

**Methods:**

We identified 131 patients with decompensated cirrhosis with an index cirrhosis-related admission between May 1, 2018, and May 1, 2019. We then examined patient portal enrollment and use data during the 6-month period preceding the study period. Portal functions evaluated included sending a message, reading a message, and reading a test result. Use was categorized as active (sending a message) and passive (reading a message or test result) and was further stratified as no, moderate, or frequent use based on the frequency of portal function use compared to the mean. The primary outcomes were 90-day and overall readmissions, adjusted for age, model for end-stage liver disease–sodium, alcohol-related cirrhosis etiology, ascites, and hepatic encephalopathy. Portal functions assessed included sending a message, reading a message, and reading a result; the total number of times a portal function was performed was divided by the number of months the patient was enrolled in the patient portal during the 6-month period.

**Results:**

The study population was 50.4% (66/131) female, with a mean age of 58 years. Enrollment in the patient portal was 63.4% (83/131), and there was no significant difference in enrollment based on clinical or demographic characteristics. For the entire cohort, 14.5% (19/131) and 22.1% (29/131) of patients were moderate and frequent active users, respectively. Of those enrolled in the patient portal, 97.6% (81/83) of patients were moderate or frequent passive users for both reading a message and reading a test result. Moderate active users had less 90-day readmissions (odds ratio 0.77, 95% CI 0.60-1.00) and overall readmissions (subdistribution hazard ratio 0.42, 95% CI 0.21-0.84), compared to nonactive users. There was no relationship between readmissions and passive use.

**Conclusions:**

Passive use of the patient portal is very high but is not associated with the risk of readmissions in people with decompensated cirrhosis. However, moderately active use of the patient portal is associated with a reduced risk of readmissions. Further work is needed to identify possible confounders and refine key use behaviors that may be protective with regard to the risk of readmission in this population.

## Introduction

The 90-day readmission rates in persons with cirrhosis range from 21% to 53% [1,2]. Many readmissions may be prevented through augmented ambulatory care and monitoring [3]. Interventions to decrease the burden of liver disease have focused primarily on increasing outpatient monitoring through the use of early patient follow-up after hospitalization and specialized clinics [4]. More recently, there have been efforts to use telehealth tools with a specific focus on asynchronous care and remote patient monitoring [5-7]. Indeed, telehealth interventions have increased substantially since the pandemic, are viewed favorably by patients with cirrhosis [8,9], and are likely cost-effective [10]. However, most of these telehealth interventions require the creation and implementation of a novel tool with inherent barriers to widespread use. Patient portals, however, are an existing electronic medical record telehealth tool that allow patients to view their test results, review clinical documentation, request medication refills, and communicate with their health care team through the exchange of messages [11]. Patient portals are widespread across institutions and are perhaps the most common telehealth tool available to and used by patients with cirrhosis [8,9], thus lending themselves as a telehealth tool that can be leveraged toward addressing gaps in the clinical care of persons with cirrhosis.

Although portal use has been characterized in multiple chronic conditions, data are lacking regarding both the patterns of patient portal use in persons with cirrhosis and whether portal activity modifies clinical outcomes. Herein, we characterize patient portal use in patients with decompensated cirrhosis and evaluate its association with cirrhosis-related readmissions.

## Methods

### Recruitment

In this retrospective study, we leveraged a large cohort of patients with cirrhosis to identify 131 patients with chart review–confirmed decompensated cirrhosis followed in the hepatology clinic at the University of Michigan Medical Center (a large tertiary care center) who had an index cirrhosis-related admission between May 1, 2018, and May 1, 2019, at our center. Decompensated cirrhosis was defined as having a history of any of the following cirrhosis-related complications: hepatic encephalopathy, ascites, variceal hemorrhage, spontaneous bacterial peritonitis, hepatic hydrothorax, hepatopulmonary syndrome, or hepatorenal syndrome. Cirrhosis-related admission was defined as an admission related to ascites (or hepatic hydrothorax) or volume management, kidney injury, infection, gastrointestinal bleeding, jaundice, and hepatic encephalopathy. Patients were excluded if they were aged 75 years or older; there was a lack of at least 1 outpatient hepatology appointment in the 3 months preceding May 1, 2018; there was the occurrence of liver transplantation within 3 months after the index hospitalization; if death occurred during or within 1 week of the index admission; or if the patient was discharged to hospice after index admission.

### Patient Portal Use Characteristics

Patient portal enrollment and use were assessed for each patient during the 6 months preceding the study period (November 1, 2017, to May 1, 2018). Portal functions assessed included sending a message, reading a message, and reading a result; the total number of times a portal function was performed was divided by the number of months the patient was enrolled in the patient portal during the 6-month period. Consistent with previous studies, active portal use was defined as sending a message, and passive portal use was defined as reading a message or a result [11-13]. The use of a portal function was further stratified into frequent use (use equal to or above the mean), moderate use (any nonzero use below the mean), and nonuse (zero use, including those without patient portal enrollment).

### Statistical Analysis

The primary outcome was the odds of a 90-day readmission, which was analyzed using logistic regression. The secondary outcome was the overall risk of readmission during follow-up based on active use, and this was assessed using Fine and Gray competing risk regression with death or liver transplantation as a competing event. A priori, multivariable models were adjusted for age, model for end-stage liver disease–sodium (MELD-Na), alcohol-related cirrhosis, ascites, and hepatic encephalopathy.

### Ethical Considerations

This study was approved by the University of Michigan’s institutional review board (HUM00172327). A waiver of informed consent was provided as this retrospective study used existing information. Privacy and confidentiality of all patient data were maintained throughout the entire study. No compensation was provided to patients as this study only involved the review of retrospective data.

## Results

The study population (N=131) was 50.4% (66/131) female, 91.6% (120/131) White, with a mean age of 58 years and a mean MELD-Na of 14.3. Nonalcoholic fatty liver disease (47/131, 35.9%) and alcohol (42/131, 32.1%) were the most common etiologies of cirrhosis, and ascites (110/131, 84%) and hepatic encephalopathy (91/131, 69.4%) were the most frequent decompensations. A total of 10 patients had a history of hepatocellular carcinoma at inclusion. The most common cirrhosis-related reasons for index admission were hepatic encephalopathy (41/131, 31.3%), infection (41/131, 31.3%), and ascites (24/131, 18.3%). A total of 63.4% (83/131) of patients were enrolled in the patient portal. There was no statistically significant difference between those enrolled and those not enrolled in the patient portal based on age, sex, race, MELD-Na, history of ascites, or history of hepatic encephalopathy. In terms of active use, 63.4% (83/131) of patients were nonusers, 14.5% (19/131) were moderate users, and 22.1% (29/131) were frequent users of the patient portal. Within passive use (reading a message or test result), 41.2% (54/131) and 42% (55/131) of patients were moderate users, and 20.6% (27/131) and 19.8% (26/131) of patients were frequent users, respectively.

During the 90 days after the index admission, 47% (62/131) of patients experienced a readmission, with hepatic encephalopathy being the most common indication. A total of 5 patients died or had a liver transplantation during this period and were excluded from the analysis. For the portal function of sending a message, those categorized as moderate users were significantly less likely to experience a 90-day readmission (odds ratio [OR] 0.77, 95% CI 0.60-1.00) compared to nonusers (Table 1). There was no difference in the risk of 90-day readmission between frequent active users (OR 0.93, 95% CI 0.75-1.17) and nonusers. No relationship was found between the frequency of passive portal use (reading a message or a test result) and the risk of 90-day readmission.

**Table 1:** Frequency of portal use function and 90-day and overall readmission.

Variable^a^		90-day readmissions, adjusted^b^ OR^c^ (95% CI)	Overall readmissions, adjusted SHR^d^ (95% CI)
**Sent messages**			
	Nonuse (reference)	—^e^	—
	Moderate use	0.77 (0.60-1.00)	0.42 (0.21-0.84)
	High use	0.93 (0.75-1.17)	1.08 (0.71-1.65)
**Read messages**			
	Nonuse (reference)	—	—
	Moderate use	1.01 (0.82-1.23)	0.95 (0.58-1.54)
	High use	0.88 (0.69-1.12)	1.00 (0.65-1.54)
**Read results**			
	Nonuse (reference)	—	—
	Moderate use	0.94 (0.77-1.15)	0.74 (0.47-1.17)
	High use	0.96 (0.75-1.23)	0.98 (0.61-1.59)

^a^Retrospective analysis (May 1, 2018, to May 1, 2019; University of Michigan Medical Center) evaluating the association between the frequency of patient portal use (sent messages, read messages, and read results) and the risk of 90-day and overall readmissions in patients with decompensated cirrhosis.

^b^Models were adjusted for age, model for end-stage liver disease–sodium, alcohol-related cirrhosis, ascites, and hepatic encephalopathy.

^c^OR: odds ratio.

^d^SHR: subdistribution hazard ratio.

^e^N/A.

During follow-up after the index admission, the first event experienced by 75.6% (99/131) of patients was a readmission, 12 (9.2%) underwent liver transplantation or died as their first event, and the remainder (20/131, 15.3%) experienced no event. Moderate active portal use was associated with a 58% reduction in the risk of readmission (adjusted subdistribution hazard ratio 0.42, 95% CI 0.21-0.84), but not frequent active portal use.

## Discussion

### Overview

We found that a majority of patients (83/131, 63.3%) with decompensated cirrhosis at our center were enrolled in the patient portal during the study period. While overall active portal use was modest (48/131, 36.6%), the proportion of patients engaging in passive use was very high (81/83, 98% patients enrolled in the portal). Our findings show that moderate active communication through the portal (sending messages) was associated with decreased odds of a 90-day readmission and a reduced risk of overall readmissions in decompensated cirrhosis. Readmissions were not modified by patterns of passive use.

### Portal Use and Clinical Outcomes

Previous studies examining the association between patient portal use and clinical outcomes in other disease states are mixed. Indeed, in a study by Griffin et al [11], active portal use (light use and frequent use) was associated with an increased risk of 30-day readmission after an index hospitalization for an acute myocardial infarction, congestive heart failure, or pneumonia. In a study examining patient portal use during an admission, in-hospital use did not modify the risk of 30-day readmissions [14]. Conversely, there is evidence for improved glycemic control [12] and adherence to cancer screening [15] through engagement in the patient portal.

One possible explanation for our findings is that moderate active interaction may facilitate clinical monitoring and improve patient self-efficacy, while passive use lacks the ability to provide health care teams with the feedback necessary to make real-time adjustments to clinical care plans. Frequent, high use is perhaps either a marker of impending readmission, unproductive communications, or failure to accurately assess or provide an appropriate solution for the condition. Additionally, patient portal message content characteristics are nuanced and encompass messages that are both health-related (health information sharing) and health-unrelated (requests for paperwork and expressions of gratitude) [16,17]. Future studies should assess how various types of communication impact clinical outcomes in cirrhosis. Similarly, not all communication is effective communication to address a disease-related need, and more work is needed to understand the key aspects of cirrhosis-related care that can be effectively facilitated by patient portal interactions.

### Limitations and Future Directions

First, the content of the messages and subspecialties of the message recipients were unknown and may have confounded the results. Communication performed through the patient portal messages is diverse and includes both clinical and nonclinical content. Studies that build on this work should closely examine how the interplay between the frequency of portal use and the thematic content of the messages associates with clinical outcomes. Second, interactions with the patient portal are likely variable over time and may change after an admission. However, we reduced this limitation by establishing an average use of key portal features in the 6-month period before the study period. Due to the study cohort averages used to define portal use intensity, our findings may not be generalizable across populations. Third, we were not able to determine whether portal use was a surrogate for another patient-specific characteristic not identified, such as digital health literacy and technology acceptance [9]. Engagement with digital health tools may be related to social determinants of health, including broadband access, smartphone and laptop ownership, and educational attainment [18,19]. Fourth, given there is no accepted definition to classify portal use intensity, it is possible that our method introduced bias in how patients were grouped. Incorporating into the analysis the use of other patient portal functionalities (log-ins, refill requests, and review of clinical documentation) and clustering use into groups using statistical methodologies may provide a more robust assessment of clinical outcomes associated with portal use [20]. Finally, this study was based on data from the pre–COVID-19 era, and both patient portal enrollment and use substantially increased during the pandemic.

### Conclusion

Active use of the patient portal may be a helpful tool in addressing the inherently high readmission rates in cirrhosis. However, additional work is needed to identify possible confounders and refine key use behaviors of both patients and providers that may be protective or maladaptive in relation to the risk of readmission.

## Abbreviations

MELD-Na: model for end-stage liver disease–sodium
OR: odds ratio

## Data Availability

The data sets generated during and/or analyzed during this study are not available.
